# A Sneak Peek Toward Polyaryletherketone (PAEK) Polymer: A Review

**DOI:** 10.7759/cureus.31042

**Published:** 2022-11-03

**Authors:** Labdhi M Maloo, Sumeet H Toshniwal, Amit Reche, Priyanka Paul, Mayur B Wanjari

**Affiliations:** 1 Department of Conservative Dentistry and Endodontics, Sharad Pawar Dental College and Hospital, Datta Meghe Institute of Medical Sciences (Deemed to be University), Wardha, IND; 2 Department of Public Health Dentistry, Sharad Pawar Dental College and Hospital, Datta Meghe Institute of Medical Sciences (Deemed to be University), Wardha, IND; 3 Department of Research, Jawaharlal Nehru Medical College, Datta Meghe Institute of Medical Sciences (Deemed to be University), Wardha, IND

**Keywords:** bioactivity, implant material, polyetherketoneketone polymer, polyetheretherketone polymer, polyaryletherketone polymer

## Abstract

Metals, acrylics, zirconia, and other such materials have been conventionally used in dentistry. The development of polymers has facilitated significant changes in clinical dentistry. High-performance polymer materials are at the forefront of dentistry. Polyaryletherketone (PAEK) is a biocompatible polycyclic, aromatic, thermoplastic polymer having good mechanical and thermal properties. It has two members: polyetheretherketone (PEEK) and polyetherketoneketone (PEKK). The difference in the ratio and structure of ether to ketone group affects the melting point and glass transition temperature. PEEK and PEKK have a high impact with their physical and mechanical characteristics similar to that of the bone and mimic the natural tooth structure. It can be used as a substitute for metals and other materials owing to its non-allergic properties and acceptable aesthetics. Currently, to modify the properties of both materials, additives were used. This semicrystalline structure does not provide any kind of mutagenicity and cytotoxicity. This review provides insight into the properties and applications of polymer in dentistry and the medical field as well. There is room for metal-free restorations in modern dental practice due to the rising demand for aesthetics, a few disadvantages with existing materials, and clinicians changing their paradigms toward metal-free restorations. The objective of this review is to provide a thorough understanding of PEEK and PEKK and their multiple uses in prosthetic, implant frameworks, abutments, crowns, and ortho wires, as well as in restorative dentistry, while demonstrating their potential for clinical applications.

## Introduction and background

In order to accomplish the ideal, dentistry has been actively evolving its methods and utilizing advanced materials. Metals, acrylics, zirconia, and other such materials have been conventionally used in dentistry. The development of polymers has facilitated significant changes in clinical dentistry [1]. It has great mechanical and physical properties, and it is biocompatible in nature [2]. In search of newer materials and to overcome the limitations of materials used in day-to-day life, more advanced materials are introduced; one of them is polyaryletherketone (PAEK). Since the 1980s, PAEK is used in the engineering field showing great machinability [3]. It is a thermoplastic polymer and performs extraordinarily well with its effective mechanical and chemical resistance [4]. High-performance polymer materials are at the forefront of dentistry research because they might enhance framework properties and possibly lower rehabilitation costs.

PAEK is a semicrystalline thermoplastic polymer; it has a glass transition temperature of about 157°C and a melting temperature of 370°C [5]. PAEK polymer contains ether and ketone groups. The difference in the ratio and structure of ether to ketone group affects the melting point and glass transition temperature. The more the ketone group, the more the polarity and rigidity and thus the more the glass transition temperature and melting point. PAEK has two family members; the first is polyetheretherketone (PEEK), the monomer unit of ether ether ketone, and the other one is polyetherketoneketone (PEKK), the monomer unit of ether ketone ketone. Polymers with semicrystalline structure do not provide any kind of mutagenicity and cytotoxicity [2-5]. PEEK and PEKK have a high impact with their physical and mechanical characteristics similar to that of the bone.

Additionally, it was discovered that PEEK has biocompatible qualities [2]. The combination of positive in vivo and in vitro results made polymer very popular for medical uses such as orthopedic and social implants, though its potential for dental applications has also been studied for over a decade. Its use has been recommended for a variety of intraoral fixed and removable prosthesis and restoration. It can also be used as ortho wires because it might exert more favorable forces than standard wires [6]. Recently, PEKK, which is also a biocompatible high-performance polymer (Bio-HPP), was shown as an innovative dental material. It is a potential replacement to metal and glass ceramics in dental application because of its fracture resistance, improved stress distribution, and shock-absorbing properties. Its aforementioned characteristics make it highly compatible in the medical industry. Though one of the organic polymers of PAEK family is commonly used as implant material, it has been acknowledged as a suitable substitute for long-established titanium in ortho application [3]. The objective of this review is to provide a thorough understanding of PEEK and PEKK and their multiple uses in prosthetic, implant frameworks, abutments, crowns, and ortho wires, as well as in restorative dentistry, while demonstrating their potential for clinical applications [2].

## Review

**Figure 1:**
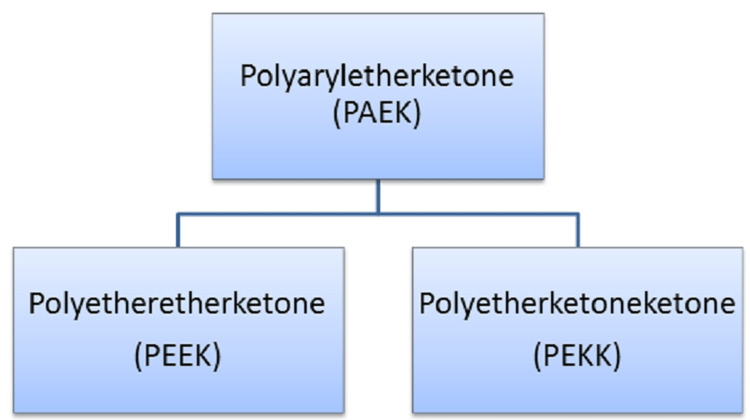
Showing two family members of PAEK

PAEK, a semicrystalline polycyclic, aromatic, thermoplastic polymer, has five family members. This polymer has monomers and differs in ratio and structure of ether to ketone group: polyetherketone (PEK), polyetheretherketone (PEEK), polyetherketoneketone (PEKK), polyetheretherketoneketone (PEEKK), and polyetherketoneetherketoneketone (PEKEKK). PEEK and PEKK are polymers of PAEK family that can be used widely in dentistry (Figure 1). Further article shows a detailed review of PEEK and PEKK including structure, synthesis, properties, and applications.

Structure and synthesis of PEEK and PEKK

**Figure 2:**
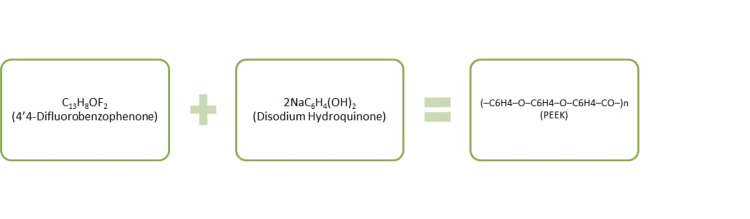
Synthesis of PEEK PEEK: polyetheretherketone Source: [8]

**Figure 3:**
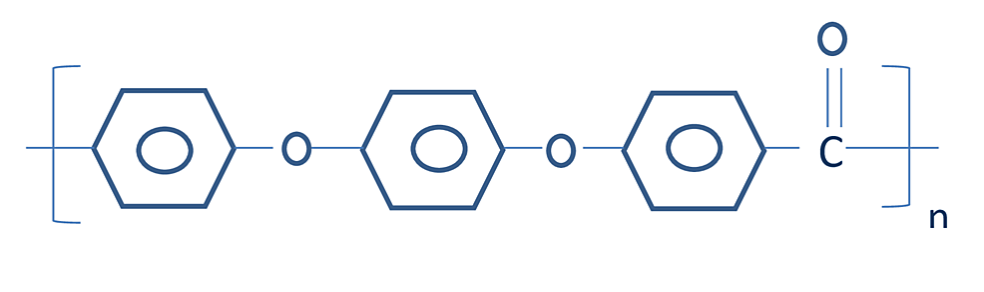
Structure of PEEK polymer PEEK: polyetheretherketone

PEEK is a semicrystalline thermoplastic, polycyclic, aromatic polymer. When 4,4'-difluorobenzophenone is added with disodium hydroquinone at temperature of 300°C and melting point of 335°C, PEEK is formed (Figure 2 and Figure 3) [7]. The manufacturing of PEEK polymer is done by injection molding, compression molding, computer-aided design/computer-assisted manufacture (CAD/CAM), or rapid prototyping.

**Figure 4:**
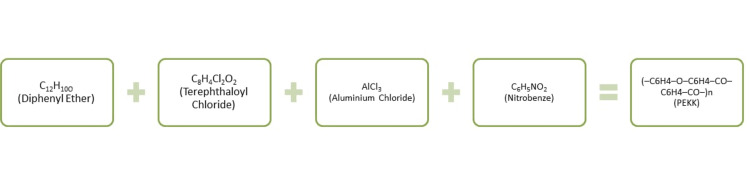
Synthesis of PEKK PEKK: polyetherketoneketone

**Figure 5:**
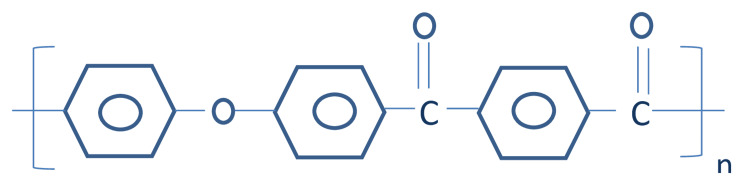
Structure of PEKK polymer PEKK: polyetherketoneketone

PEKK is an ultra-high-molecular-weight polymer with linear aromatic polyether ketone group introduced by Bonner in the year 1962 [9]. PEKK is a product of diphenyl ether and terephthaloyl chloride in which aluminum chloride and nitrobenzene are added (Figure 4 and Figure 5). Compression molding and particle leaching are such process used by Converse et al. for developing a PEKK [10]. It differs from PEEK in the presence of extra ketone group in its aromatic ring, which increases polarity and backbone rigidity of the material. PEKK has a melting point of 305°C [2].

Properties of PEEK and PEKK

PAEK polymer shows various enhanced physical, mechanical, and biological properties (Table 1) [2,11-15].

**Table 1:** Properties of PEEK and PEKK polymer PEEK: polyetheretherketone; PEKK: polyetherketoneketone; VHN: Vickers hardness number; FEFF1.3: free energy force field 1.3

Properties	PEEK	PEKK
Physical properties	Biocompatible, minimal plaque affinity, aesthetics, dimensional stability, good polishability, and wear resistance	Biocompatible, shock absorbance, aesthetics, flexibility, microporosity, and hydrophilic nature
Mechanical properties		
Tensile strength (MPa)	100.69	115
Elastic modulus (GPa)	3.5	5.1
Flexural strength (MPa)	163.88	200
Compressive strength (MPa)	118-169	246
Melting temperature (°C)	334-350	363-386
Hardness	26-29 VHN	252 MPa
Water absorption (mg/mm^3^)	0.1-0.5	8.7
Density (g/cm^3^)	1.3	FEFF1.3
Biological properties	Osteointegration property	Osteointegration property and antibacterial

Applications of PEEK and PEKK

There are several applications of PEEK and PEKK due to its various benefits in dentistry. It can be used widely as implants, prosthetic material, ceramic filler, and so on. Detailed explanatory benefits of PEEK and PEKK are shown further.

Implant Material

PEEK is a material that can be utilized as implants. Titanium is relentlessly used as an implant material, but due to some of its limitations, PEEK has been introduced as such; titanium is metal, whereas PEEK is metal-free material. Titanium is heavier, and PEEK is lighter in weight; also, titanium causes hypersensitivity as it is a metal. The osseointegration property of PEEK is good, but PEEK material alone does not have certain enhancing properties. It requires certain surface modifications to increase its osseoconductive property significantly [16,17]. The best surface treatment for polymer is carbon fiber reinforced (CFR), which increases its modulus of elasticity nearer to cortical bone [18]. According to documentation, the elastic modulus of PEEK is 3.6 GPa, but after reinforcing it with carbon fibers, elastic modulus increases to 18 GPa. The elastic modulus of cortical bone is 15 GPa. In comparison with elastic modulus of titanium that is 110 GPa, the elastic modulus of PEEK is close to the elastic modulus of the bone [17,19,20]. PEEK also has flexural strength of 140-170 MPa [17]. Many modifications are done with PEEK polymer such as spin coating, that is, covering a PEEK surface with nanoparticles of calcium hydroxyapatite, and get incorporated into the implant surface creating a better bone implant contact [21]. Another method for CFR-PEEK is by plasma spraying with titanium oxide and hydroxyapatite crystals; then, immersing it in alpha tricalcium phosphate shows better properties, and this was currently given by Nakahara et al. [22]. It is also modified with tantalum ions to form Ta_2_O_5_ nanoparticles, which provides better osteoconductivity, elasticity, and attachment with cortical bone, in which PEEK limits as it is bioinert in nature [23]. PEEK can also be modified by processing it with chemicals by amination, sulfonation, and nitration in pre-polymerization or post-polymerization [7]. Sulfonation is one of the methods incorporated with PEEK polymer for preventing the formation of biofilm, in return making it *Staphylococcus aureus* and *Escherichia coli* resistant [24]. Due to mastication or any parafunctional habits such as bruxism, titanium abutment or screws are replaced by PEEK as it does not cause hypersensitivity unlike titanium and can bear forces such as titanium [25].

PEKK is another member of PAEK family that also shows better physical, mechanical, and biological characteristics. It is also used as an alternative to titanium in implants because it shows better compatibility with other materials. PEKK already has better elastic modulus than PEEK; even surface treatment is not much required. But to make it more effective, additives with PEKK were introduced. Sulfonation was done with PEKK; also, it was observed that PEKK was having early healing of the bone and good osteointegration property compared to sulfonated PEEK. The combination of PEKK and titanium is used to provide retention for a very long period of time [26]. The combination of PT40, that is, PEKK and tantalum with 40 v%, is also used, as the ability of PT40 to bear load and serve as bony substitute is excellent [27]. After temporalis myofascial flap surgery, hollowing of the bone was replaced by temporal implants achieving smooth healing process, and patients found it to be aesthetically pleasing [28]. PEKK offers better retention when inserted in overdentures. Other than dentistry, PEKK is also used as orthopedic implants [29].

Prosthetic Material

Metals such as cobalt-chromium and titanium were previously utilized as prosthesis; however, they are aesthetically unacceptable, heavy, and metallic in taste, and some people are allergic to it. PEEK polymer was introduced to use it as a prosthetic material in removable partial dentures (RPDs) and fixed partial dentures (FPDs), crowns, and so on. In removable prosthesis, it can be used as clasps and can replace cobalt-chromium alloys [30]. Obturators are also prosthetic devices that are generally made up of acrylic, but the superior properties of PEEK can overcome these [31]. For people who are partially edentulous, RPDs involve replacing teeth and other structures with artificial ones. When cobalt-chromium, titanium alloy, and PEEK were compared, PEEK was more useful for the protection of periodontal fibers, as well as distributed masticatory force evenly all over the denture base [32]. However, some studies are still going on to prove the ability of the polymer. CAD/CAM is the smart advance for making digital impression and to reduce chairside time. Enhanced biocompatibility, the elimination of allergic reactions, durability, and elasticity, as well as being metal-free, make it more aesthetically appealing, and economical advantages would be made possible by combining polymer-based materials and digital fabrication techniques [33]. Polymethylmethacrylate (PMMA) is used to make FPDs machined with CAD/CAM, but PEEK is the different material that can be used as an alternative to PMMA. Fracture resistance of PEEK is higher than those of lithium disilicate glass-ceramic (950N), alumina (851N), and zirconia (981-1331N); also, the abrasive property of PEEK is fantastic [34-37]. PEEK shows resistance to chemical and radiation as well, to prevent damage from glass and carbon fibers [38]. PEEK with nanoparticles of zirconia is another modified form that shows low wear and friction resistance. Nanoparticles such as titanium dioxide, fluorapatite, and hydroxyapatite were added to PEEK in order to increase their elastic recovery, antibacterial property, stability, and biocompatibility [39,40].

PEEK is a better material than metals, but PEKK shows superior shock absorbance and less stress concentration of 49 MPa, whereas PEEK shows higher stress on the base of prosthesis of 58 MPa [41]. The lesser the stress concentration, the lesser the fracture risk on the denture base. PEKK is the topmost member of PAEK family and the resultant of amorphous and semicrystalline structure. PEKK has lower crystallization rate, and it is less affected by cooling when heated in lower temperature, so irrespective of CAD/CAM and conventional denture-making procedures, it can be machined by printers with a low build chamber temperature of less than 200°C [42-44]. Retention is the most important factor for dentures. In removable prosthesis, clasps made up of PEKK can be used for longer duration and also can be used as an alternative to nylon inserts as it is more retentive and abrasive [45]. Acrylic resin shows poor marginal fit after a decade, which causes plaque retention, recurrent caries, periodontal problems, and prosthetic failure. CAD/CAM-based digital technique with PEKK polymer along with surface modifications makes higher retentive and perfectly fitted denture. Thus, PEKK coping was proved better than zirconia coping [46,47]. PEKK is also used as obturator such as in removable speech bulb prosthesis [48]. PEEK and PEKK polymers can be used in making crowns and veneers. Another enhanced form of PEEK is biocompatible high-performance polymer (Bio-HPP) with the addition of ceramic filler of 20% with size of 0.3-0.5 μm in resin matrix, given by Bredent in Germany [49]. Bio-HPP PEEK has excellent polishing capabilities, low plaque affinity, nonmetallic taste, and strong wear resistance, which are all characteristics that make it anti-allergic [50]. PEEK and PEKK can be used with jiffy dentures to produce interim dentures or intermediate dentures.

Endodontic Material

Whenever more than 50% of the tooth structure is lost and the tooth cannot resist torsional stress, post and core is used. There are two types of posts: metallic and nonmetallic. Titanium, stainless steel, and gold alloys are metals used to fabricate post; due to increasing demands of aesthetics and allergic reactions of patients to metal, metallic posts have been disregarded. Other types of post are nonmetallic such as zirconia, ceramic, carbon fiber, and glass fiber. These materials have their certain limitations such as high modulus of elasticity, brittleness, and leaving voids on the surface; exposure to moisture will lead to failure and can cause root fracture. To overcome all limitations of such materials, a low-elastic-modulus, higher-strength, tissue-compatible material of PAEK family was smartly made. In endodontically treated tooth, PEEK polymer can be used as a post material as it will provide less stress on the tooth structure, as well as in the core material [51].

For restoration purpose, as PEEK is bioinert, the addition of hydroxyapatite makes it bioactive and hydrophilic in nature [52]. In order to prove the ability of fracture resistance of PEEK polymer, a comparative study was done between polymer-infiltrated ceramic, fiber-reinforced composite post, and PEEK. Higher fracture resistance was shown by PEEK polymer among all aesthetic post material [53]. Since the PEEK material's elastic modulus is so near to that of dentin tissue, less stress development in the tooth and after core restoration than in conventional post systems was observed [54]. For irrigation during root canal treatment, tips used to irrigate the canals can be made from PEEK polymer as it is chemical resistant and does not react with irrigants such as sodium hypochlorite, chlorhexidine, and ethylenediaminetetraacetic acid [14,55].

PEKK and polymer other than PEEK can also be used as endodontic posts and endo crowns [56,57]. It also possess similar properties of that of PEEK in relation with the fabrication of endodontic post. PEKK has modulus of elasticity similar to that of dentin and mimics natural tooth structure. PEKK has low modulus of elasticity and flexural strength compared with other metallic and nonmetallic conventional post materials, which helps in less stress distribution and dispersion of stress in intraradicular space in order to prevent the fracture of root surface [56]. These also require ferrule of at least 1.5 mm to be present for fracture resistance. There are two methods of fabrication of post: prefabrication and custom-made. When compared to prefabricated PEKK posts, custom-made PEKK posts showed stronger bonds [57].

Orthodontic Material

Materials usually used for ortho wires and brackets are acrylic, metals, or ceramics. PEEK and PEKK are the materials that can replace metal ortho wires and provide proper force normally required. Other than metal, ceramic brackets are also used as brackets for aesthetic purpose. Ceramic brackets have some shortcomings in relation with properties such as strength, and the force applied by brackets made up of ceramic is comparatively less, which in turn increases the treatment span. To overcome this limitation, PEEK is a more feasible material that can be used. CAD/CAM, which is a new digital technology, when combined with PEEK polymer can be used as an alternative to conventional self-cure and heat cure space maintainers, making it favorable and aesthetically pleasing for the patient [58]. Besides aesthetics, metal ortho wires, brackets, and appliances have certain limitations. For example, metal releases nickel and chromium ions. When metal wire and brackets are coupled, they undergo galvanic reaction and corrosion. PEEK wire is a material that shows promise in terms of stain resistance. 16-22 PEEK and 19-25 PEEK had greater load reductions than nickel-titanium (Ni-Ti). Intentionally, 70% and 80% of the initial load was kept, and the preserved amount was adequate for orthodontic treatment. Thus, PEEK can be used as an alternative to metal for better orthodontic treatment [59]. The novel PEEK tube also demonstrated a good balance of aesthetic and practical qualities along with other orthodontic appliances. Wire is easily incorporated in the hole of the tube because of its elasticity and flexibility [60].

Difference between PEEK and PEKK

PAEK polymer family members PEEK and PEKK have similar properties except for some that are mentioned in Table 2.

**Table 2:** Difference between PEEK and PEKK PEEK: polyetheretherketone; PEKK: polyetherketoneketone

PEEK	PEKK
Contains two ether group and one keto group	Contains two keto group and one ether group
Higher extrusion temperature (almost 400°C)	Lower extrusion temperature (340°C-360°C)
Lower glass transition temperature (143°C)	Higher glass transition temperature than PEEK (159°C)
Low modulus of elasticity than PEKK	Greater modulus of elasticity than PEEK
As PEEK has low modulus of elasticity, it requires surface treatment	It works if surface treatment is not done with PEKK polymer
80% straight and 20% kinked segments melt at 360°C	60% straight and 40% kinked segments melt at 305°C

## Conclusions

The polymer-based material PAEK is a polycyclic, thermoplastic, semicrystalline structure with superior properties. PEEK and PEKK are polymers that can be used widely in dentistry as implants, RPDs, FPDs, crowns, ortho wires, and so on. Other than dentistry, it can be used in the medical field also. These materials benefit not only clinicians but also patients. As it is metal-free and has an elastic structure, it differs from normal metallic substances used in day-to-day life. Currently, to modify the properties of both materials, additives are used. In light of this review, practicing this material in various procedures can provide improvised and beneficiary treatment options. Various studies are still going to prove the efficacy of this polymer.
